# Novel Molecular Subtypes and Related Score Based on Histone Acetylation Modification in Renal Clear Cell Carcinoma

**DOI:** 10.3389/fcell.2021.668810

**Published:** 2021-09-23

**Authors:** Shichao Wang, Ting Xiang, Ling Yu, Junmao Wen, Fang Liu, Dong Yang, Wei Wu, Ling Hu

**Affiliations:** ^1^Department of Cardiology, The First Affiliated Hospital of Guangzhou University of Chinese Medicine, Guangzhou, China; ^2^Guangzhou University of Chinese Medicine, Guangzhou, China; ^3^Department of Traditional Chinese Medicine, The First Affiliated Hospital of Sun Yat-sen University, Guangzhou, China; ^4^Department of Traditional Chinese Medicine, The First Affiliated Hospital of Guangdong Pharmaceutical University, Guangzhou, China

**Keywords:** renal clear cell carcinoma, molecular subtype, score, immunotherapy, histone (de)acetylation

## Abstract

**Background:** Histone acetylation modification has been found to be correlated the development of renal carcinoma; however, its role in clear cell renal carcinoma (ccRCC) remains to be investigated. Thus, this study aimed to identify the molecular subtypes and establish a relevant score based on histone acetylation modification in ccRCC.

**Methods:** Gene expression and mutation data were retrieved from The Cancer Genome Atlas database. Molecular subtypes were identified by unsupervised clustering based on histone acetylation regulators expression, and the molecular and clinical characteristics including survival, tumor microenvironment, gene set variation, immune cell infiltration, and immune checkpoints in each subtype were investigated. Next, we employed univariate Cox analysis to analyze these genes and established acetylation-related score by lasso regression analysis. Furthermore, we investigated the differences including survival, signaling pathways, mutational landscape, and tumor mutation burden (TMB) between high-risk and low-risk groups. The established score was validated by receiver operating curve and univariate and multivariate Cox regression analyses. We also established a nomogram including acetylation score, age, gender, grade, and stage and verified it by decision curve analysis and calibration plot. The E-MTAB-1980 cohort from the ArrayExpress database was employed as a reference to validate the established score.

**Results:** Thirty-three types of histone acetylation regulators were employed in this study, and two clusters were identified. The two clusters presented significant differences in survival, tumor microenvironment, immune cell infiltration, immune checkpoints, and signaling pathways. Furthermore, an acetylation-related score, composed of six genes (BRD9, HDAC10, KAT2A, KAT5, BRDT, SIRT1, KAT6A, HDAC5), was verified to be significantly associated with prognosis and TMB. Thus, the established scores were successfully verified by the validated cohort, and the nomogram was constructed and successfully validated.

**Conclusion:** The identification of the histone acetylation-related subtypes and score in our study may help reveal the potential relation between histone acetylation and immunity and provide novel insights for the development of individualized therapy for ccRCC.

## Introduction

As one of the most common malignant urological tumors, renal cell carcinoma (RCC) accounts for approximately 2–3% of adult tumors and 90% of kidney cancers (Moch et al., 2016). At least 350,000 new cases of RCC occur worldwide and more than 140,000 patients die of this disease each year (Siegel et al., 2020). According to the pathologic classification, RCC is generally divided into four pathological subtypes: clear cell renal carcinoma (ccRCC), granulosa cell renal carcinoma, mixed cell renal carcinoma, and undifferentiated cell renal carcinoma. ccRCC is the major subtype in RCC, which accounts for 70–80% (Moch et al., 2016). Notorious for the insidious onset (Jonasch et al., 2014) and insensitivity to traditional chemoradiotherapy, the incidence and metastatic rate of ccRCC are still high. Although the molecular targeted therapy presents the remarkable curative effectiveness in advanced ccRCC, the drug response rate and obvious side effect limits the clinical benefit (Siegel et al., 2020). Consequently, the investigation and development of prognostic biomarkers are urgently needed in ccRCC.

Epigenetic aberrations, comprising several different aberrations such as changes in histone modifications, DNA methylation, and microRNA levels, are commonly found in RCC, which indicates that epigenetic reprogramming plays a crucial role in RCC development (Joosten et al., 2018). In terms of histone modification, histone demethylases (KDMs) act as a central role in histone modification. The emerging evidences supported that KDMs such as KDM3A, KDM5C, KDM6A, and KDM6B play important roles in RCC, and KDMs could promote RCC development and progression via hypoxia-mediated angiogenesis pathways (Guo and Zhang, 2017). It has been reported that the epigenetic aberrations such as DNA methylation and histone modifications (acetylation and methylation) can significantly contribute to the transcriptomic upregulation of immune checkpoints and their ligands (Saleh et al., 2020). There were also many inhibitors such as the histone deacetylation inhibitor, histone methyltransferase inhibitor, and histone demethylase inhibitor developed in epigenetic therapy for RCC (Mehdi and Riazalhosseini, 2017). These findings have constructed a promising therapeutic modality using the combination of epigenetic and immunotherapeutic agents. Therefore, the potential mechanism among epigenetic modification and immunotherapy in ccRCC still remain to be explored.

In this research, we identified novel molecular subtypes based on the gene expression of histone acetylation regulators. The two clusters (acetylation or deacetylation cluster) present notable differences in clinical and immunologic features, including survival, gene mutation, signaling pathways, immune cell infiltration, and immune checkpoints expression. Interestingly, we observed that the deacetylation cluster exhibited worse prognosis and the lowered immune cell infiltration. Furthermore, we established the acetylation-related score and validated its prognostic value in clinic. We believe that the established subtypes not only help in elucidating the underlying association linking histone acetylation modification and immunotherapy in ccRCC but can also promote the development of individualized clinical treatments.

## Materials and Methods

### Data Collection

Gene expression and mutation data, and clinicopathological messages were obtained from The Cancer Genome Atlas (TCGA) database^1^ and the ArrayExpress database.^2^ KIRC cohort from the TCGA database (training dataset) and E-MTAB-1980 cohort from the ArrayExpress database (validation dataset) were employed in our research. According to the previous study (Favazza et al., 2017), we selected the patients with VHL mutation, copy number loss for chromosome 3p, or both. Furthermore, we identified 57 histone modification-related genes, including 24 genes (*KDM1A, KDM6B, KDM6A, KDM4A, KDM5B, KDM2A, KDM5A, KMT2D, KMT5A, KMT2A, SETD2, NSD1, SMYD3, NSD2, DOT1L, EZH2, SETD7, CARM1, SUV39H1, EHMT2, ATRX, EED, PC, RAG2*) related to methylation and 33 genes (*HDAC1, HDAC2, HDAC3, HDAC4, HDAC5, HDAC6, HDAC7, HDAC8, HDAC9, HDAC10, HDAC11, SIRT6, SIRT1, SIRT3, SIRT7, SIRT2, SIRT5, SIRT4, KAT2A, KAT6A, KAT6B, CREBBP, KAT2B, KAT5, KAT7, EP300, KAT8, BRD2, BRD9, BRD4, BRDT, BRD7, BRD3*) related to acetylation from the previous studies (Audia and Campbell, 2016; Gong et al., 2016; Hammond et al., 2017).

### Landscape and Consensus Clustering for Histone Acetylation Regulators

Considering the functional difference between methylation and acetylation in histone modification, we performed the single sample gene set enrichment analysis (ssGSEA) and survival analysis to further selection. The ssGSEA is a special type of GSEA that can estimate a score for each case by the ‘‘GSVA’’ package. The cases in the KIRC cohort were divided into two groups (high-score or low-score group) based on the median of scores. We compared the survival difference between two groups using the ‘‘survival’’ package and found the significant correlation between acetylation score and survival, so we selected the acetylation-related genes for further investigation. The correlation among the gene expression of 33 acetylation-related genes was investigated by the ‘‘corrplot’’ package. The expression difference of included genes between tumor and normal groups was explored using the Wilcoxon rank sum test. In addition, the summary of somatic mutation and copy number variations from 33 acetylation-related genes was generated by the cBioPortal website.^3^ To further investigate the distinct histone acetylation modification pattern in renal carcinoma, we classified the patients based on the expression of included genes by the “ConsensusClusterPlus” package. The number of clusters and their stability was determined by a consensus clustering algorithm; 1,000 repetitions were performed to guarantee the stability of the subtypes. The “ConsensusClusterPlus” function with the parameter “pItem = 0.8, pFeature = 1, clusterAlg = km, distance = euclidean” was applied in our study.

### Difference Features Between Acetylation-Related Subtypes

After confirming the clusters, a series of analyses was applied to validate the novel molecular subtypes. Considering the different functional genes (acetylation or deacetylation) in histone acetylation modification, we defined the cluster (acetylation or deacetylation) by heatmap and ssGSEA. Next, principal component analysis was performed to display the distribution of samples. Moreover, to explore the time-dependent prognostic value of the subtypes, survival analysis was executed by the “survival” package. Meanwhile, to investigate the different tumor microenvironment (TME) between subtypes, we estimated the stromal/immune score and tumor purity of each case using the “ESTIMATE” package. The “ESTIMATE” package was utilized to predict tumor purity, as it estimates the presence of infiltrating stromal/immune cells in TME (Yoshihara et al., 2013). The “estimate score” represents the total score of immune and stromal score, and it is in inverse proportion to tumor purity. The ESTIMATE algorithm is executed by ssGSEA and finally generates three scores: the stromal score (indicates the presence of stromal cells in tumor tissues), the immune score (represents the infiltration of immune cells in tumor tissues), and the tumor purity. Furthermore, to explore the different biological processes between established subtypes, gene set variation analysis (GSVA) was performed by the “GSVA” package. GSVA is usually executed to compare the difference in the pathway and biological process activity in samples from an expression dataset (Hänzelmann et al., 2013). The gene sets of ‘‘h.all.v7.1.symbols’’ were downloaded from the MSigDB database^4^ for GSVA analysis. Results with a p-value of less than 0.05 were considered to be statistically significant.

### Immune Cell Infiltration and Immune Checkpoint Expression Between Two Subtypes

We investigated the immune cell infiltration between established subtypes based on the ‘‘TIMER’’ and ‘‘MCP-counter’’ methods. TIMER^5^ is a comprehensive resource for the immune cell infiltration, which estimated six types of immune cells (B cells, CD4+ T cells, CD8+ T cells, neutrophils, macrophages, and dendritic cells). MCP-counter estimates the abundance of 10 cell populations, including T cells, CD8+ T cells, cytotoxicity score, NK cells, B cells, monocytes, macrophages, dendritic cells, and neutrophils. Furthermore, immune checkpoint genes (20, encoding both ligands and receptors) were retrieved from a previous study (Burugu et al., 2018).

### Establishment of Acetylation-Related Score

To identify the prognostic genes of histone acetylation in KIRC, we performed univariate Cox regression analysis. The genes with a *p*-value <0.01 in univariate analysis were eligible for further analyses. The lasso regression analysis was applied to establish the acetylation-related score by “glmnet” and “survival” package. In this analysis, a lasso penalty was used to account for shrinkage and variable selection. The optimal value of the lambda penalty parameter was defined by performing 10 cross-validations. The lambda was calculated using the “glmnet” function with the parameter “family = cox, maxit = 1000.” The calculation formula for acetylation-related score was as follows:


score=⁢(coefficientmRNA1×expressionofmRNA1)+(coefficientmRNA2×expressionofmRNA2)+

⋯+(coefficientmRNAn×expressionmRNAn)

According to the median of the established score, cases were divided into two groups (high-risk or low-risk group). Survival analysis was performed based on this grouping strategy. To further verify the acetylation-related score, a receiver operating characteristic (ROC) curve was constructed to examine the prognostic accuracy. Besides, we performed GSEA to further explore the significantly enriched pathways between groups. GSEA is a computational method that identifies whether a previously defined set of genes shows statistically significant differences between two biological states (Subramanian et al., 2005). In the GSEA software, the number of permutations was set to 10,000 and the permutation type was phenotype. The max size of excluded larger sets was 500 and the min size was 15. The most relevant pathways were identified based on the normalized p-value and enrichment score. Finally, univariate and multivariate Cox regression analyses were performed to validate whether the acetylation-related score could be an independent prognostic marker in ccRCC.

### Correlation Between Mutation and Acetylation-Related Score

To further compare the mutational features, we investigated the difference of TMB and mutational landscapes between two clusters. The tumor mutational burden (TMB) was defined as the total number of errors in somatic gene coding, base substitution, gene insertions, or deletions detected in every million bases. To calculate the TMB in each case, the total number of mutations counted was divided by the exome size (38 Mb was utilized as the exome size). TMB correlation analysis and survival analysis was performed to explore the associations between TMB and the subtypes. We also investigated the somatic gene mutations in the different subtypes by the “maftools” package.

### Nomogram Construction and Validation

Considering the clinical application of acetylation-related score, the nomogram was constructed based on Cox regression model. The nomogram included age, gender, grade, stage, and acetylation-related score. Decision curve analysis was performed to compare the net benefits of different models (stage, grade, acetylation-related score, and nomogram). The concordance index, calibration plot, and ROC curve were used to verify the nomogram. Model performance was evaluated through calibration and discrimination (Alba et al., 2017). Bias-corrected calibration for 3 and 5 years of overall survival rate was performed by 1,000 bootstrap resamples to evaluate the consistency between the observed and estimated survival probability by the “rms” package. The calibration was calculated by the “calibrate” function with the parameter “cmethod = KM, method = boot, m = 80.” Discrimination was evaluated by Harrell’s concordance index (C-index) and ROC curve. A higher area under curve (AUC) value revealed superior model discriminative ability, and a higher C-index value demonstrated better model-fitting performance (Zhang et al., 2020). Decision curve analysis (DCA) was further performed to measure and compare the clinical utilities of the different prognostic models. DCA is a method for evaluating the benefit of a diagnosis test across a range of patient preferences for accepting risk of undertreatment and overtreatment to facilitate decisions about test selection and use (Fitzgerald et al., 2015).

### Score Validation

Here, we employed the E-MTAB-1980 cohort from the ArrayExpress database for score validation. Survival analysis, ROC curve, and univariate and multivariate Cox regression analyses were also performed to estimate the clinical value of acetylation-related score.

## Results

### Landscape of Genetic Variation and Expression of Histone Acetylation Regulators in KIRC

**FIGURE 1:**
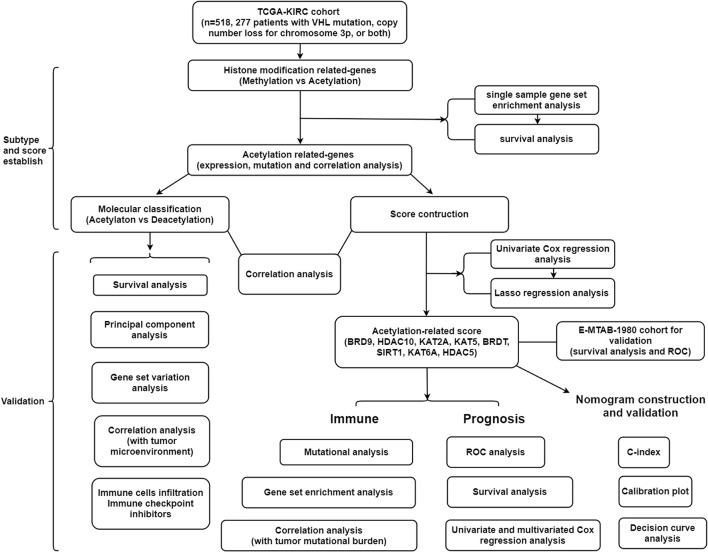
The flowchart of this study.

**FIGURE 2:**
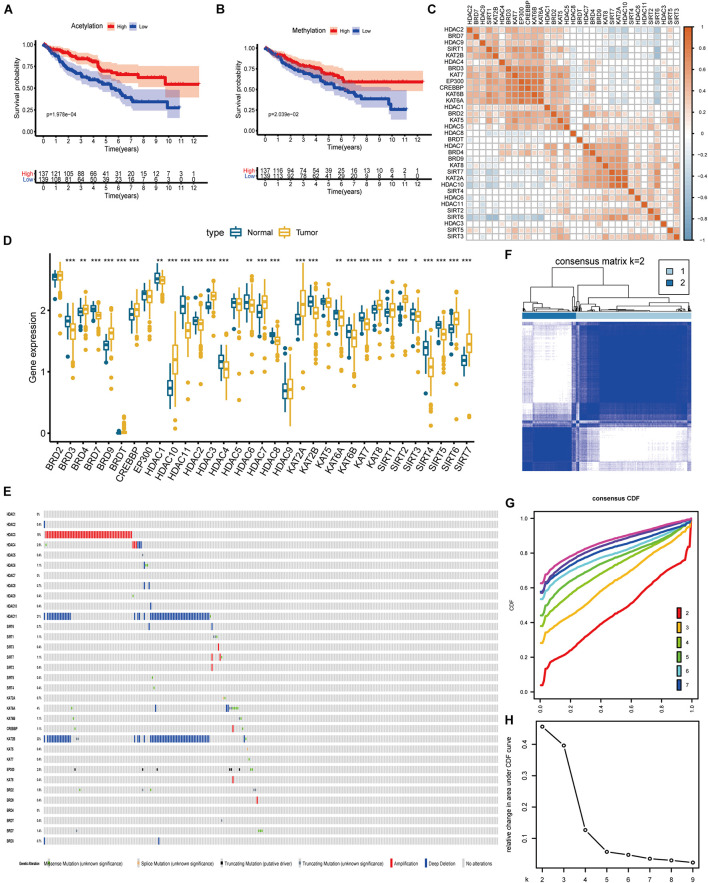
The landscape of histone acetylation regulators in ccRCC and subtype identification **(A)** Survival analysis between high and low score of acetylation based on ssGSEA. **(B)** Survival analysis between high and low score of methylation based on ssGSEA. **(C)** Correlation among 33 histone acetylation regulators. **(D)** The results of differentially expressed analysis from histone acetylation regulators. *p < 0.05, **p < 0.01, ***p < 0.001. **(E)** Data on the somatic mutation and copy number variations of 33 histone acetylation regulators. Panels **(F–H)** show the most appropriate value for subtype identification.

A summary of this research is shown in the form of a flowchart in Figure 1. The clinical details of the patients employed in our research are summarized in Table 1 and Supplementary Material. As illustrated in Figures 2A,B, the acetylation-related genes exhibited the much significant prognostic value than methylation. Therefore, we supposed that acetylation-related genes were more valuable in KIRC and employed them for further analyses. The correlations of 33 acetylation regulators are presented in Figure 2C. It was found that the histone acetylation regulators not only exhibited a remarkable interaction in the same functional category but also showed a significant correlation among different functional categories. The comparisons of gene expression between tumor and normal groups from Figure 2D demonstrate that significant expression difference was found in most of regulators (27/33). A summary of the incidence of somatic mutations and copy number variation of 33 acetylation regulators is presented in Figure 2E. The abovementioned results reveal that the imbalance and cross-talk among acetylation regulators may play a crucial role in KIRC.

**TABLE 1:** Baseline patient characteristic in the two cohorts.

Clinical characteristics		Number	Percent (%)
**TCGA-KIRC (*n* = 518)**			
Survival status	Survival	353	68
	Death	165	32
Age	≤65 years	339	65.4
	>65 years	179	34.6
Gender	Female	181	35
	Male	337	65
Stage	I	259	50
	II	55	10.6
	III	122	23.6
	IV	82	15.8
Grade (5 patients missing)	G1	14	2.7
	G2	221	43.1
	G3	204	39.8
	G4	74	14.4
T classification	T1	265	51.2
	T2	67	12.9
	T3	175	33.8
	T4	11	2.1
M classification	M0	413	79.7
	M1	77	14.9
	MX	28	5.4
N classification	N0	238	45.9
	N1	15	2.9
	NX	265	51.2
**3E-MTAB-1980 (*n* = 99)**			
Survival status	Survival	76	76.8
	Death	23	23.2
Gender	Female	23	23.2
	Male	76	76.8
Age	≤65 years	56	56.6
	>65 years	43	43.4
Grade	G1	13	13.1
	G2	59	59.6
	G3	22	22.2
	G4	5	5.1
T classification	T1	68	68.7
	T2	9	9.1
	T3	21	21.2
	T4	1	1
M classification	M0	87	87.9
	M1	12	12.1
N classification	N0	92	92.9
	N1	3	3
	N2	4	4.1

### Identification of Acetylation-Associated Molecular Subtypes

**FIGURE 3:**
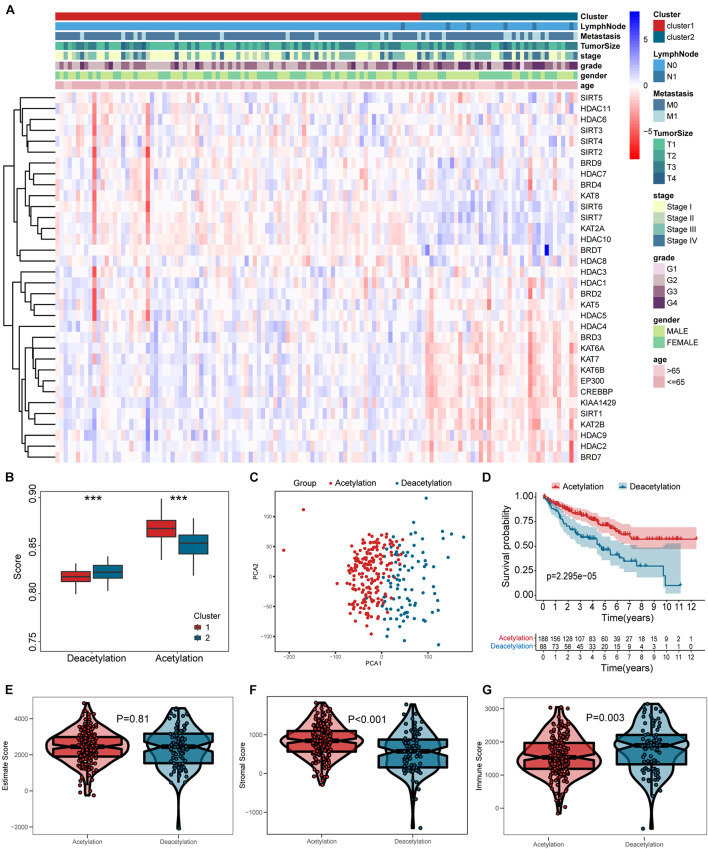
Heatmap and verification of histone acetylation-related molecular subtypes. **(A)** The heatmap including clusters, clinical parameters, and acetylation-related genes. **(B)** The definition for cluster based on ssGSEA. **(C)** Results of PCA. **(D)** Survival analysis. **(E–G)** Comparisons of estimate score, stromal score, and immune score between two subtypes, respectively. ***p < 0.001.

The results from Figure 2F indicate that a remarkable difference is observed between the two clusters while k value is equal to 2. Figures 2G,H shows that the relative change is remarkable between 2 and 3. Consequently, the cases were divided into two clusters, including 189 cases in cluster 1 and the remainder in cluster 2. As shown in the heatmap (Figure 3A), no specific functional feature is found in cluster 1 or cluster 2, so we further employed the ssGSEA to define the clusters. The results from Figure 3B demonstrate that cluster 1 presents the significantly obvious feature of acetylation while cluster 2, deacetylation. Consequently, cluster 1 is defined as the acetylation cluster and cluster 2 is the deacetylation cluster. The results of PCA, shown in Figure 3C, indicate that the cases from each cluster could be distinguished visually. Survival analysis for the two clusters demonstrates that the deacetylation cluster exhibits a survival disadvantage in overall survival (Figure 3D).

### Different Immunologic Features in Subtypes

**FIGURE 4:**
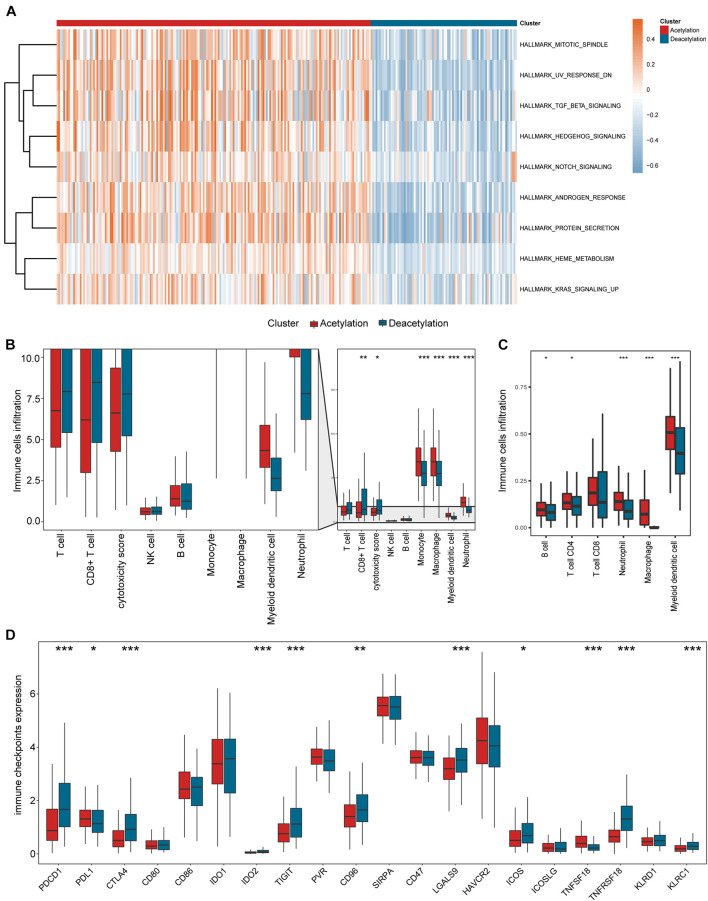
Relevant signaling pathways, immune cell infiltration, and immune checkpoints in subtypes. **(A)** The results of gene set variation analysis. **(B,C)** Comparisons of immune cell infiltration between two subtypes. **(D)** Comparison of immune checkpoints expression between two subtypes. In panels **(B–D)**, *p < 0.05, **p < 0.01, ***p < 0.001.

According to the results in Figures 3E–G, the deacetylation cluster presented the lower stromal score and higher immune score than the acetylation cluster, which indicates that two clusters present the different TME. Meanwhile, the different biological processes are also found between two clusters (Figure 4A). Subsequently, we compared the immune cell infiltration between two clusters and found that the deacetylation cluster presents the significantly lower immune cell infiltration in monocytes, macrophages, dendritic cells, and neutrophils (Figures 4B,C). We also observed that some immune checkpoints (*PDCD1, CTLA4, IDO2, LGALS9, ICOS, TNFRSF18*, and *KLRC1*) present significantly higher expression in the deacetylation cluster while others (*PDL1* and *TNFSF18*), in the acetylation cluster (Figure 4D).

### Construction of Acetylation-Related Score

**FIGURE 5:**
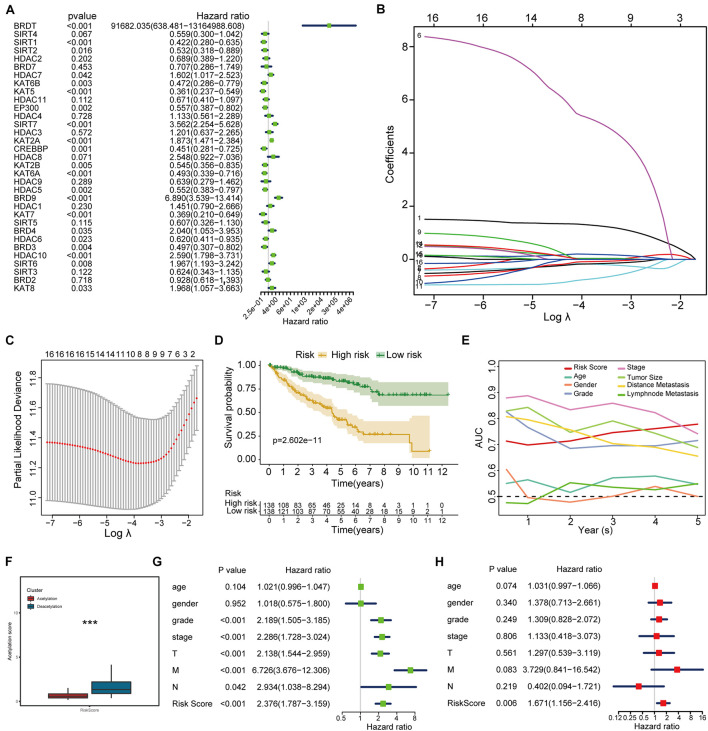
Histone acetylation-related score construction and validation. **(A)** Results of univariate Cox regression analysis. **(B,C)** Results of lasso regression analysis. **(D)** Survival analysis between high-risk and low-risk groups. **(E)** ROC analysis including established score and other clinical parameters from 1 to 5 years. **(F)** Correlation between established subtypes and acetylation-related score. **(G,H)** The results of univariate and multivariate Cox regression analysis. ***p < 0.001.

To further investigate the prognostic value of histone acetylation regulators in KIRC, we employed the univariate Cox analysis to select the genes. The results of univariate Cox analysis (Figure 5A) demonstrate that 16 genes (*BRDT, SIRT1, KAT6B, KAT5, EP300, SIRT7, KAT2A, CREBBP, KAT2B, KAT6A, HDAC5, BRD9, KAT7, BRD3, HDAC10, SIRT6*) are eligible for lasso regression analysis (*p* < 0.01), and the results of lasso regression analysis from Figures 5B,C confirmed the score composed of eight genes, namely, *BRD9*, *HDAC10*, *KAT2A*, *KAT5*, *BRDT*, *SIRT1*, *KAT6A*, and *HDAC5*. The higher score exhibits the worse prognosis in survival analysis (Figure 5D). Furthermore, the acetylation-related score presents the highest AUC value in 5 years (Figure 5E), which shows its potential predictive performance in clinic. Besides, the deacetylation cluster also showed the higher acetylation-related score in Figure 5F. Moreover, the results of univariate and multivariate Cox regression analysis (Figures 5G,H) indicated that the acetylation-related score may serve as an independent prognostic marker in KIRC.

### Different Mutation Features Between High-Risk and Low-Risk Groups

**FIGURE 6:**
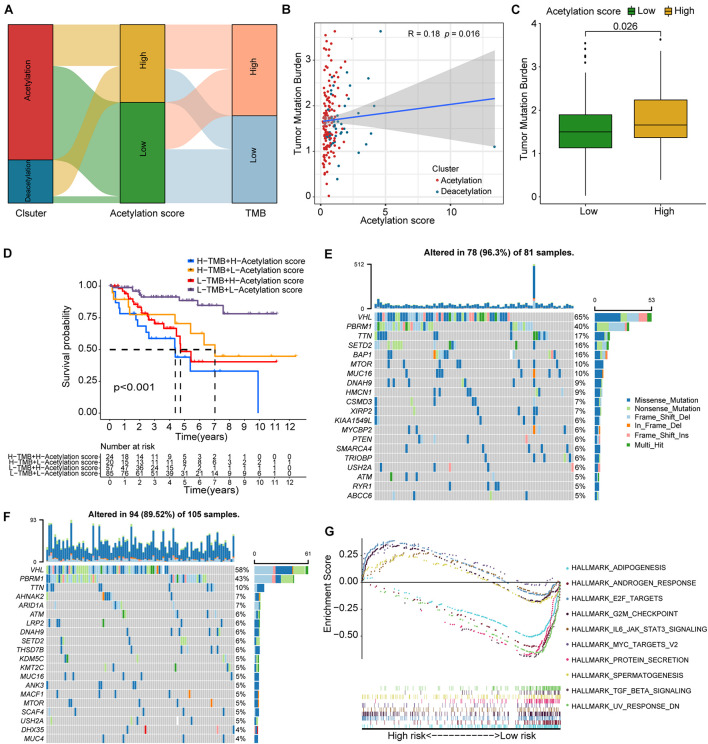
TMB correlation, mutation landscape, and GSEA. **(A)** Sankey plot including subtypes, acetylation-related score, and TMB. **(B)** Correlation analysis between TMB and acetylation-related score. **(C)** Comparison of TMB between high and low acetylation-related score groups. **(D)** Survival analysis for the combination of TMB and acetylation-related score. **(E,F)** The waterfall plots of somatic mutation for high-risk and low-risk groups respectively. **(G)** The results of GSEA.

To further understand the prognostic difference between high-risk and low-risk groups, we investigated tumor mutation burden and somatic mutational landscape between two groups. The Sankey plot from Figure 6A shows the interaction among molecular subtypes, acetylation-related score, and TMB, and the significant correlation is found between acetylation-related score and TMB (Figure 6B). At the same time, higher acetylation-related score exhibits the higher TMB in Figure 6C. Interestingly, we combined the TMB and acetylation-related score and found that the patients with higher TMB and higher acetylation-related score presented the worst prognosis (Figure 6D). In terms of somatic mutation, the high-risk group presents the higher mutational rate than the low-risk group (Figures 6E,F), and the high-risk group significantly enriched in the pathways of MYC targets, E2F targets, G2M checkpoint, IL6 JAK STAT3 signaling, and spermatogenesis, while in the low-risk group, protein secretion, androgen response, adipogenesis, TGF beta signaling, and UV response (Figure 6G).

### Nomogram Construction and Validation

**FIGURE 7:**
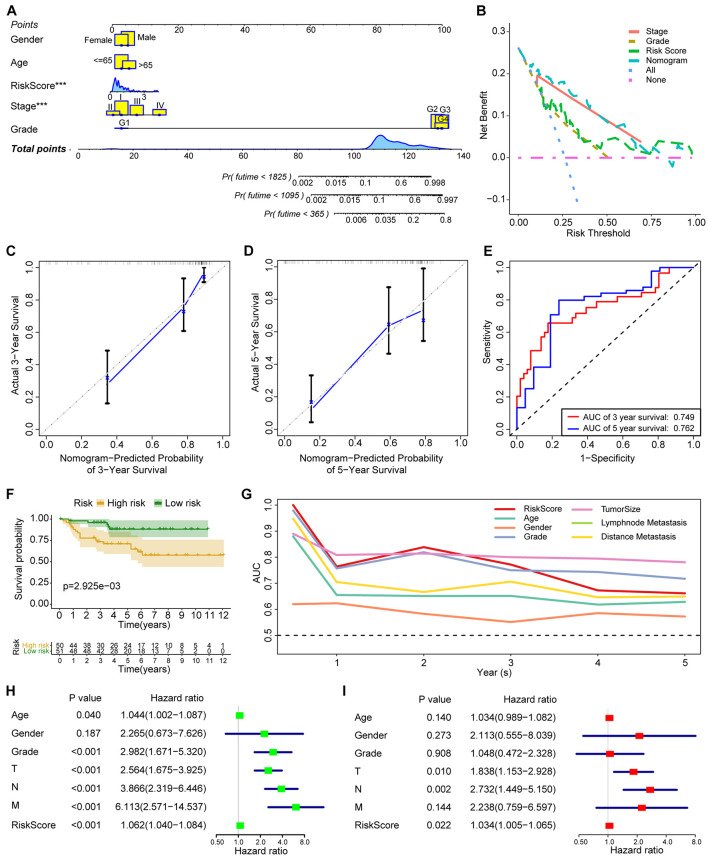
The construction and validation of nomogram and the validation of acetylation-related score. **(A)** The nomogram including age, gender, grade, stage, and acetylation-related score. **(B)** The results of decision curve analysis. **(C,D)** Calibration plot for 3 and 5 years. **(E)** ROC analysis for 3 and 5 years. **(F)** Survival analysis of acetylation-related score from validation dataset. **(G)** ROC analysis from validation dataset. **(H,I)** The results of univariate and multivariate Cox regression analysis from validation dataset. ***p < 0.001.

As demonstrated in Figure 7A, a nomogram including age, gender, grade, stage, and acetylation-related score is constructed. Decision curve analysis (Figure 7B) demonstrated that the nomogram model exhibited a higher net benefit than the other models. The concordance index of the nomogram was 0.83, and the calibration plot for the probability of survival at 5 years (Figures 7C,D) showed no obvious inconsistency between the nomogram predictions and real observations. ROC analysis (Figure 7E) indicated that the nomogram exhibited moderate predictive value in ccRCC.

### Verification From the ArrayExpress Cohort

To validate the established score, we employed the independent cohort (E-MTAB-1980) to perform the survival analysis. The results of survival analysis from Figure 7 showed that significant differences were found between the high-risk and low-risk group (Figure 7F), and the acetylation score also presents the third higher predictive performance in the validation cohort (Figure 7G). Finally, the results of univariate and multivariate Cox regression analysis (Figures 7H,I) also indicated that the acetylation-related score may serve as an independent prognostic marker in the validation cohort.

## Discussion

Despite the great development in tumor diagnosis and treatment, the prognosis of ccRCC patients is still unsatisfactory. The 5-year survival rate of ccRCC is above 90% at early stage but 15% in advance stage (Hsieh et al., 2017). Since the high-throughput sequencing has been developed, more and more novel predictive models have been established to improve the dilemma of poor prognosis of advanced ccRCC. For example, a prognostic signature based on RNA binding protein-related genes in ccRCC has been developed (Chen et al., 2021). Another study (Gui et al., 2021) also established an autophagy-related long non-coding lncRNA signature in ccRCC. However, these signatures are far from able to meet clinical demands and more molecular subtypes need to be identified. Emerging evidences supported that epigenetic modification especially histone modification may contribute to the upregulation of immune checkpoints and promote the treatment of ccRCC (Saleh et al., 2020). In this study, we first identified novel molecular subtypes based on histone acetylation regulators. Epigenetic regulation of gene expression occurs in the protein level (post-translational histone modifications), DNA level (DNA methylation), and RNA level (non-coding RNAs). Post-translational modification of specific amino acids of histone tails plays an important role in regulating the chromatin structure and dominating gene expression (Tessarz and Kouzarides, 2014). Post-translational modification of histone includes various types, such as acetylation, methylation, phosphorylation, ubiquitylation, and sumoylation (Kouzarides, 2007), of which lysine acetylation and methylation are the best understood. In our research, we confirmed 24 methylation-related genes and 33 acetylation-related genes and found that acetylation-related genes significantly correlated with the prognosis of ccRCC patients by ssGSEA. The imbalance and cross-talk among 33 acetylation-related genes are observed in our results, which verifies that histone acetylation modification plays a crucial role in ccRCC. Therefore, we considered that histone acetylation modification is more valuable in ccRCC and employed the related genes for further analyses.

After identifying the subtypes based on acetylation regulators by consensus cluster algorithm, we observed that two clusters (acetylation and deacetylation cluster) exhibit the different clinical and biological characteristics. The deacetylation cluster presents the worse prognosis and is highly activated in the tumor proliferation pathway, which draws our attention. Simultaneously, the deacetylation cluster exhibits the higher tumor purity than the acetylation cluster, which is consistent with poor prognosis. As the difference of TME was found between two clusters, we further investigated the immunologic features of two clusters. It is worth noting that the deacetylation cluster generally presents the lower immune cell infiltration than the acetylation cluster, which indicated that the deacetylation cluster presented the immunosuppressive TME. In terms of immune checkpoints, different clusters show various expression levels of immune checkpoints, but we found that no pair of receptor and ligand was significantly expressed in the same cluster, so the correlation between histone acetylation modification and immunotherapy in ccRCC needs further validation.

Considering the individual heterogeneity of histone acetylation modification, it was necessary to quantify the histone acetylation modification in ccRCC. Consequently, we established an acetylation-related score to evaluate histone acetylation modification in patients with ccRCC. The deacetylation cluster presents a high acetylation-related score. Although the genes involved in the established score remain to be investigated by experiments, our research provides the bioinformatic evidences of these genes for further validation.

Higher acetylation score results in the activation of tumor progression signaling pathways, and worse prognosis is consistent with the molecular characteristics of the deacetylation cluster. The acetylation-related score is validated in another independent cohort, suggesting that histone acetylation modification is a reliable tool for a comprehensive assessment of ccRCC. Considering the potential association between histone acetylation modification and immune regulatory, we further explored the correlation between acetylation-related score and TMB. It has been reported that TMB could be employed to predict the efficacy of immune checkpoint inhibitors and become a useful biomarker in identifying patients who will benefit from immunotherapy (Chan et al., 2019). Our results reveal that acetylation-related score significantly correlated with TMB, and the patients with high TMB and acetylation-related score presents the worse prognosis, which reveals the underlying and indirect association between acetylation modification and immunotherapy in ccRCC.

To the best of our knowledge, this is the first study to identify histone acetylation-related subtypes in ccRCC. We found that the patients with histone deacetylation modification present the worse prognosis and immunosuppressive TME and proposed the underlying association between histone deacetylation and immunity, which may contribute to the further functional experiments. Furthermore, a greater number of histone acetylation regulators included and the comprehensive methodology employed in our research enabled the identification of a robust score, and the score exhibits the better performance in predicting the prognosis of ccRCC. However, some limitations in our study have to be pointed out. First, no pair of receptor and ligand of immune checkpoints was highly expressed in clusters, which may be attributed to the small sample size. Further investigations may help in validating the association between histone acetylation modification and immune checkpoint inhibitors. Second, no immunotherapeutic cohort of ccRCC was performed, so the correlation between histone acetylation modification and real immunotherapeutic response remains to be explored. Finally, our results were preliminary due to the use of a bioinformatic approach. More experiments and clinical trials should be performed to validate the current evidences.

In conclusion, our research indicated the crucial role of histone acetylation modification in ccRCC. The defined subtypes and established score may contribute to validate the association linking histone acetylation modification and immunity.

## Data Availability Statement

Publicly available datasets were analyzed in this study. This data can be found here: TCGA (KIRC cohort) database (https://portal.gdc.cancer.gov/) and ArrayExpress database (https://www.ebi.ac.uk/arrayexpress/).

## Author Contributions

SW and LH designed the manuscript. SW and TX wrote and completed the manuscript. LY, JW, and FL completed the data download. TX, DY, and WW completed the data analysis. All the authors approved the final manuscript, contributed to the article, and approved the submitted version.

## Conflict of Interest

The authors declare that the research was conducted in the absence of any commercial or financial relationships that could be construed as a potential conflict of interest.

## Publisher’s Note

All claims expressed in this article are solely those of the authors and do not necessarily represent those of their affiliated organizations, or those of the publisher, the editors and the reviewers. Any product that may be evaluated in this article, or claim that may be made by its manufacturer, is not guaranteed or endorsed by the publisher.

## Footnotes

1
https://portal.gdc.cancer.gov/

2
https://www.ebi.ac.uk/arrayexpress/

3
https://www.cbioportal.org/

4
https://www.gsea-msigdb.org/gsea/index.jsp

5
http://timer.comp-genomics.org
